# CD133 Antigen as a Potential Marker of Melanoma Stem Cells: *In Vitro* and *In Vivo* Studies

**DOI:** 10.1155/2020/8810476

**Published:** 2020-12-23

**Authors:** Tomasz Kloskowski, Joanna Jarząbkowska, Arkadiusz Jundziłł, Daria Balcerczyk, Monika Buhl, Kamil Szeliski, Magdalena Bodnar, Andrzej Marszałek, Gerard Drewa, Tomasz Drewa, Marta Pokrywczyńska

**Affiliations:** ^1^Chair of Urology and Andrology, Department of Regenerative Medicine, Cell and Tissue Bank, Collegium Medicum, Nicolaus Copernicus University, Sklodowskiej-Curie 9, 85-094 Bydgoszcz, Poland; ^2^Department of Plastic, Reconstructive and Aesthetic Surgery, Collegium Medicum, Nicolaus Copernicus University, Bydgoszcz, Poland; ^3^Department of Clinical Pathomorphology, Collegium Medicum, Nicolaus Copernicus University, Bydgoszcz, Poland; ^4^Department of Clinical Pathology, Poznan University of Medical Sciences and Greater Poland Cancer Center, Poznan, Poland; ^5^University of Bydgoszcz, Bydgoszcz, Poland

## Abstract

Melanoma is the most dangerous type of skin cancer. Cancer stem cells (CSCs) are suspected to be responsible for the cancer recurrence and in the consequence for cancer therapy failure. CD133 is a potential marker for detection of melanoma CSCs. Experiments were performed on the B16-F10 mouse melanoma cell line. CD133+ cells were isolated using an immunomagnetic cell sorting technique. After isolation proliferative and clonogenic potential of CD133+, CD133- and CD133+/- were evaluated. The potential of CD133+ and CD133- cells for tumor induction was conducted on C57BL/6J mouse model. Three different cell quantities (100, 1000, 10000) were tested. Tumor morphology, number of mitoses, and tumor necrosis area were analyzed. Average 0.12% CD133+ cells were isolated. Compared to CD133- and unsorted CD133+/- cells, CD133+ cells were characterized by the higher proliferative and clonogenic potential. These properties were not confirmed *in vivo*, as both CD133+ and CD133- cells induced tumor growth in mouse model. No statistical differences in mitosis number and tumor necrosis area were observed. Simultaneous detection of CD133 antigen with other markers is necessary for accurate identification of these melanoma cancer stem cells.

## 1. Introduction

Melanoma is the most dangerous type of skin cancer. About 132000 new cases of melanoma are diagnosed each year globally [1]. The highest incidence is observed in Australia; in Europe, the highest incidence is observed in Switzerland and Scandinavian countries and the lowest in Greece and Romania [2, 3]. Melanoma accounts for only 4% of skin malignancies, but it is responsible for 79% of deaths caused by those cancer types [4].

In literature, we can find different theories about cancer development; one of them includes contribution of cancer stem cells (CSCs). Hypotheses including CSCs assume that tumor mass is highly heterogenous and consists of many cells varying in their differentiation levels [5]. CSCs can lead to development, progression, and ineffective treatment of many different cancers. Therapies targeting directly this cell type can lead to complete tumor removal. CSCs are more resistant to chemotherapy compared to other cells building the tumor mass, that is why they are probably responsible for cancer recurrence [6, 7]. CSCs can be recognized by the characteristic markers; one of them is CD133 (prominin1, AC133), a cell membrane glycoprotein present on a surface of many normal progenitor and cancer cells, including melanoma [8–10]. CSCs are also called as cancer-initiating cells, which means that implantation of these cells can lead to tumor development in the place of injection. CSCs need proper environment and stem cell niche, which control their function, that is why CSCs create tumors with different efficiency after injection, depending on selected place of implantation [11, 12].

The aim of this study was isolation of CD133 positive cells (CD133+) from mouse melanoma cell line (B16-F10) and compare their morphology and growth characteristic to negative (CD133-) and heterogenous (CD133+/-) cell line. Additionally, *in vivo* experiment on a mouse model was performed in order to check if cancer melanoma cells expressing CD133 marker have different properties for tumor induction compared to population without expression of this marker (CD133-).

## 2. Material and Methods

### 2.1. B16-F10 Cell Culture

B16-F10 mouse melanoma cell line was purchased from the American Type Culture Collection (ATCC, Mannassas, VA, USA). Cells were cultured in the DMEM/Ham's F12 medium (Dulbecco's Modified Eagle Medium, HyClone, Chicago, IL, USA) supplemented with 10% fetal bovine serum (FBS, Sigma-Aldrich, Saint-Louis, MO, USA) and antibiotics (penicillin 100 U/ml, streptomycin 100 *μ*g/ml, amphotericin B 5 *μ*g/ml, Sigma-Aldrich, Saint-Louis, MO, USA), in standard conditions at 37°C in 5% CO_2_ and 98% humidity. The culture medium was changed every 2-3 days. Cells were passaged when they reached 70-80% confluence using standard 0.05% trypsin solution (Biomed, Lublin, Poland) with addition of 0.5 mM of EDTA (POCH, Gliwice, Poland). The number of cells was calculated using the trypan blue staining.

### 2.2. Isolation of CD133+ Melanoma Cells

Cells with a potential CSC phenotype were isolated using the CD133 MicroBeads Kit (Miltenyi Biotec, Bergisch Gladbach, Germany). According to the protocol after trypsinization, centrifugation, and calculation of the cell number, 300 *μ*l of buffer (containing PBS, 0.5% BSA, and 2 mM EDTA), 100 *μ*l of FcR Blocking Reagent, and 100 *μ*l of CD133 MicroBeads were added, respectively, to the final volume of 500 *μ*l (values for 1 × 10^7^ cells). After each step, cell suspensions were mixed. After incubation for 30 minutes at 4°C, 4.5 ml of the buffer was added to the prepared mixture. The mixture was centrifuged (10 minutes, 700 × g, 4°C), and obtained cell pellet was resuspended in 500 *μ*l of buffer (Miltenyi Biotec, Bergisch Gladbach, Germany). CD133 populations were isolated from average 25 × 10^7^ of B16-F10 cells.

The CD133+ cells were separated using an immunomagnetic sorting technique. Cells were applied onto separation columns placed in the magnetic field. Positive cells linked with beads retained in the columns, while CD133- cells passed through. Finally, columns were removed from the magnetic field, and CD133+ population was flushed out with appropriate amount of buffer. For further experiments, all three populations (CD133+, CD133-, and CD133+/- heterogeneous population) were examined separately.

### 2.3. Flow Cytometry

To confirm successful separation of CD133+ cells, their phenotype was examined with flow cytometry. All three populations were stained with CD133-PE antibody (clone AC141; Miltenyi Biotec, Bergisch Gladbach, Germany), according to manufacturer recommendations. Stained cells were immediately analyzed with BD FACSCanto II (BD Biosciences, USA). A minimum of 50000 events were collected, with the exception of separated CD133+ cells, where 20000 events were collected because of small number of cells. Obtained data were analyzed using FlowJo v10 (Becton, Dickinson and Company, USA).

### 2.4. Real-Time Cell Growth Analysis

Real-time cell growth analysis was performed using the X-Celligence system (Accela, Prague, Czech Republic). Cells were seeded on the E-plates 16 (Accela, Prague, Czech Republic) in two different densities (1 × 10^3^ cells/cm^2^, 4 × 10^3^ cells/cm^2^), each density on separate plate. CD133+ and CD133- were seeded in 5 repetitions and unsorted cells in 4 repetitions; two wells on each plate were filled only with a culture medium which served as a background. The cells were cultured in the standard conditions in an incubator. Cell proliferation was measured as a cell index. Cell index is a unitless parameter measured by electrodes placed on the bottom of the E-plate wells. Electrodes measure the impedance of electron flow caused by adherent cells. Impedance can be affected by parameters such as cell number, their size, and morphology. Measurements were made every 30 min. for 10 days. Cell growth curves were plotted as a dependence of cell index from the time of culture.

### 2.5. Clonogenic Assay

CD133+, CD133-, and the unsorted cells (CD133+/-) were analyzed separately. Each of the cell fraction was seeded on 6-well culture plate with 10, 100, or 1000 of cells per well. Each experiment was performed in 6 repetitions. Plates were incubated for 6 days in the standard conditions in the incubator. The medium was changed twice. After the end of experiment, the medium was removed, and 0.5% of neutral red (Sigma-Aldrich, Saint-Louis, MO, USA) was added to each well for colony visualization. Cells were incubated with the dye for 10 minutes at 37°C. After that, the neutral red was removed, and wells were washed with a PBS. The cell colonies were observed and calculated under an inverted light microscope. Results were presented as a clonogenic index which was obtained by dividing the number of colonies by the initial number of seeded cells. Three colony types were analyzed. Cells can grow in the compact and round colonies (holoclones), loose irregular colonies (paraclones), or can have intermediate features (meroclones) [13].

### 2.6. In Vivo Evaluation of CD133+ Cells Ability to Initiate Tumor

The purpose of the *in vivo* experiment was to determine the tumor initiation capacity of potential CSCs based on the expression of the CD133 surface marker. Cells were transplanted into C57BL/6J mice which are recommended recipients of B16-F10 cells. The study was performed on 78 male 6-8 weeks mice with an average body weight of 20 g. All experiments were approved by the Polish Local Ethical Commission, permission No 29/2009.

Animals were divided into 6 equal groups (13 animals in each group). CD133+ or CD133- cells with 3 different quantities (1 × 10^2^, 1 × 10^3^, and 10 × 10^3^) suspended in 0.5 ml PBS were implanted intraperitoneally during the minilaparotomy procedure. Follow-up was 6 weeks; after that time, animals were euthanized by intraperitoneal injection of sodium pentobarbital in the dose of 30 mg/kg. The ventral layers were dissected along the midline. Cancer tumors were isolated, weighed (all tumors isolated from one focus and one animal were weighted together), and placed in a 4% phosphate buffered formalin for further histopathological analysis. The material was embedded in the paraffin, cut, and stained using hematoxylin and eosin.

### 2.7. Mitosis Number

Mitotic index was evaluated by two independent investigators. Mitotic figures were counted under the light microscope in three fields of view using 40x magnification. The samples were blinded for investigators for analysis. Results were presented as an average from at least eleven independent measurements, depending on the number of tumors formed after B16-F10 CD133+ or CD133- cells implantation. All tumors were analyzed for mitotic index examination.

### 2.8. The Area of Tumor Necrosis

The area of tumor necrosis was evaluated by two independent investigators. The analysis was performed under the light microscope using 100x magnification. The samples were blinded for investigators for analysis. Results were presented as an average from at least eleven independent measurements (in percentages), depending on the number of tumors formed after B16-F10 CD133+ or CD133- cells implantation. All tumors were analyzed for necrosis area examination.

### 2.9. Statistical Analysis

The statistical differences between tested groups were calculated by Kołmogorow-Smirnow test with nonparametric methods (Mann–Whitney test for comparison of two groups and Kruskal-Wallis test for comparison of >2 groups). Data were analyzed in the IBM SPSS 23.0 (SPSS, Cracow, Poland). The significance level *p* < 0.05 was used as significant.

## 3. Results

### 3.1. Successful Isolation of CD133+ B16-F10 Cells

**Figure 1:**
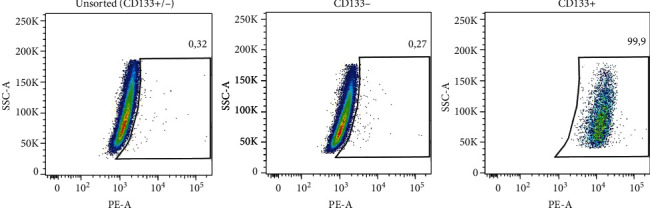
Confirmation of proper magnetic separation. Results of flow cytometry analysis of unseparated and separated cells stained with anti-CD133-PE antibody.

Magnetic-activated cell sorting (MACS) resulted in acquiring 0.12% ± 0.05%cells of the CD133+ phenotype. These results are presented as a mean from 8 different isolations. Flow cytometry analysis confirmed successful CD133+ cell isolation (Figure 1).

### 3.2. Lack of Differences in Cell Morphology between Tested Cell Populations

**Figure 2:**
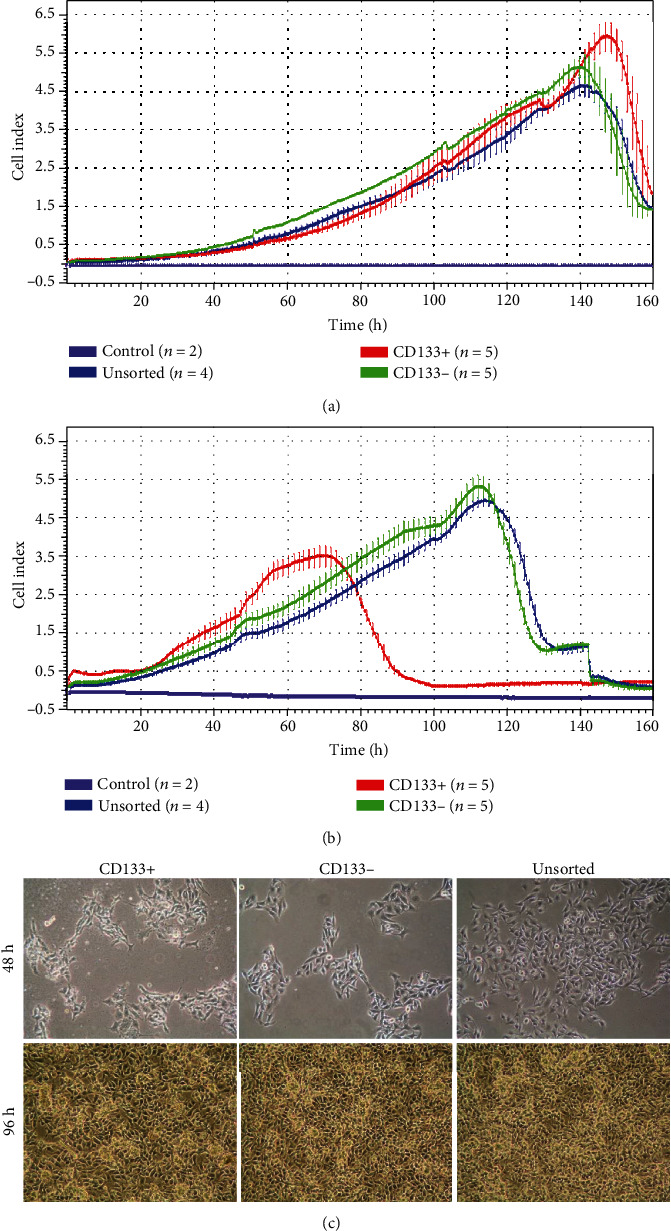
Melanoma cell characteristic after immunomagnetic cell sorting: (a, b) growth comparison of three cell populations using real-time cell analysis seeded with different cell densities: 1 × 10^3^ cells/cm^2^ (a) and 4 × 10^3^ cells/cm^2^ (b); (c) cell morphology of CD133 positive (CD133+), negative (CD133-), and unsorted (CD133+/-) B16-F10 cells after 48 h and 96 h of culture.

Microscopic observations showed no differences in B16-F10 cell morphology after 24, 48, 72, and 96 hours between the analyzed populations (CD133+, CD133-, and CD133+/-). After 48 hours, cell growth was the same in all analyzed groups. Within four days, all cultures reached 100% confluence (Figure 2(c)).

### 3.3. Different Growth Pattern of CD133+ Compared to CD133- and Unsorted B16-F10 Cells

B16-F10 CD133+, CD133-, and CD133+/- cells seeded at density of 1 × 10^3^/cm^2^ initially showed a uniform logarithmic growth (Figure 2(a)). However, from the 2nd day, slightly faster growth of CD133- and unsorted cells (CD133+/-) compared to CD133+ cells was observed. In the 3rd and 4th days of the experiment, only small differences in the cell growth were observed between tested populations. Cells reached maximum growth peak simultaneously, approximately on the 6th day of observation. Statistically significant differences in the logarithmic growth phase of cells seeded at density of 1 × 10^3^/cm^2^ were showed between CD133+ cells and the CD133- and heterogeneous CD133+/- cell populations (*p* < 0.05).

Real-time analysis of the cell growth seeded at density of 4 × 10^3^/cm^2^ showed that all tested populations initially proliferated at the same level (Figure 2(b)). On the second day, CD133+ cells began to proliferate faster, and on the 3rd day, they reached the stationary phase (plateau), while CD133- and unsorted CD133+/- cells continued to show a logarithmic growth, reaching stationary phase on the 5th day. Faster growth of CD133+ cells resulted in earlier entry into the stationary phase and decrease in proliferation probably due to detachment of cells. No differences in the cell growth were observed between the CD133- and the unsorted CD133+/- cells. In the logarithmic growth phase of cells seeded at a density of 4 × 10^3^/cm^2^, statistically significant differences between CD133+ cells and other tested populations were shown (*p* < 0.05).

### 3.4. B16-F10 CD133+ Population Generates More Colonies Number and the Highest Rate of Holoclonal Colonies Formation

**Figure 3:**
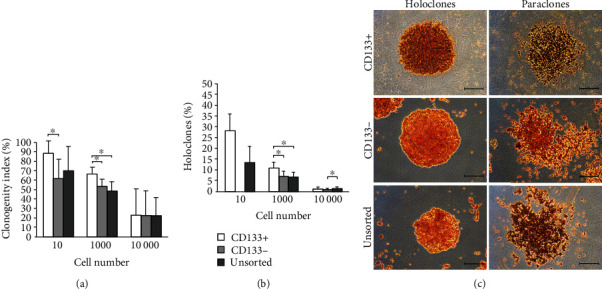
Clonogenity assay: (a) clonogenity index depending on the B16-F10 cell population (CD133+, CD133-, CD133+/-) and the cell number (*p* < 0.05); (b) percentage of holoclones generated by tested cell populations (*p* < 0.05); (c) holoclones and paraclones morphology depending on the phenotype of the tested cells, inverted microscope, bar = 400 *μ*m.

Clonogenic potential depending on the phenotype of cells and their seeding density is shown in Figure 3. The largest number of colonies was observed in the population of cells expressing the CD133 marker. The highest colony formation rate from single cell (88.3%) was found in the CD133+ population. There were statistically significant differences between CD133+ and CD133- cells (*p* < 0.05). For groups seeded with 1 × 10^2^ of cells, statistically significant differences in the number of created colonies were observed between cells showing the presence of CD133 antigen and other populations. The lowest clonogenicity potential was observed in groups in which 1 × 10^3^ of cells were seeded. However, differences between number of colonies were no statistically significant (*p* = 0.566).

Colonies with the holoclonal morphology were formed in each cell population except for CD133- cells seeded using the lowest cell number (10 cells) (Figure 2(c)). Only 1.9% and 1.3% of the colonies were holoclones in unsorted CD133+/- cell cultures and CD133-, respectively. CD133+ cells showed the highest rate of the holoclonal colony formation (2.3%). Statistically significant differences between groups were found (*p* = 0.047).

### 3.5. Lack of Statistical Significance in Tumor Formation between CD133+ and CD133- B16-F10 Cells

Palpable tumors began to appear after 9 days in mice implanted with 1 × 10^4^ cells and after 12 days in mice implanted with 1 × 10^3^ cells. After 14-17 days, a tumor development led to death of 15 animals (including 8 in the CD133+ group and 7 in the CD133- group) after injection of 1 × 10^3^ cells and 18 animals (including 10 in the CD133+ group and 8 in the CD133- group) after injection of 1 × 10^4^ cells. After 20-22 days, tumor was lethal for 2 mice from 1 × 10^2^ CD133+ group and 2 from 1 × 10^2^ CD133- group. Other animals survived the 6-week follow-up (Table S1). The highest number of induced tumors was found in the group of mice treated with 1 × 10^4^ cells (12/13 individuals (92%) in the CD133+ group and 10/13 (77%) in the CD133- group). Tumors quickly reached large sizes, growing at the site of implantation, and quickly led to death of animals. In the groups treated with 1 × 10^3^ cells, neoplastic process occurred in 9/13 (69%) mice implanted with CD133+ population and 7/13 (54%) with CD133- population. These mice also showed the most advanced cancers, with numerous metastases to the kidneys, liver, and diaphragm. In the other groups, the tumors were most common in the peritoneal cavity, at the implant site, and metastatic lesions were less numerous. The lowest morbidity occurred in the groups with the lowest number of implanted cells (1 × 10^2^), and it was 7/13 (54%) for the CD133+ group and 5/13 (38%) for the CD133- group. Tumors in these groups reached large sizes but grew slowly and did not caused rapid death of animals. The total number of tumors was about 27% higher in the CD133+ group (133 vs. 105; *p* = 0.490). In the case of survival rate, about 15% more mice completed the follow-up in the CD133- group (*p* = 0.651).

We decided to analyze all animals from one group together, regardless of survival rate, because higher mortality indicates on significant progress of disease. Additionally, combination of all animals from each group allowed to receive appropriate number of animals for statistical analysis.

The number of developed tumors was compared between the groups (*n* = 78, Table S1). Injection of CD133+ cells initiated the emergence of 56 tumor foci in the recipient organisms, about 22% (*p* = 0.190) more than after injection of the CD133- fraction (46 tumor foci).

Tumors were located at the site of cell administration, in the peritoneal cavity (in 47 subjects), diaphragm (17), kidneys (15), liver (14), seminal vesicle (7), and in the lungs (3).

Regardless of the number of implanted cells, a potential to tumorigenesis initiation was higher by about 15% (*p* = 0.238) in the population of cells expressing the CD133 marker in comparison to the population of CD133- cells. There were no statistically significant differences, but a similar trend was observed in all groups.

**Figure 4:**
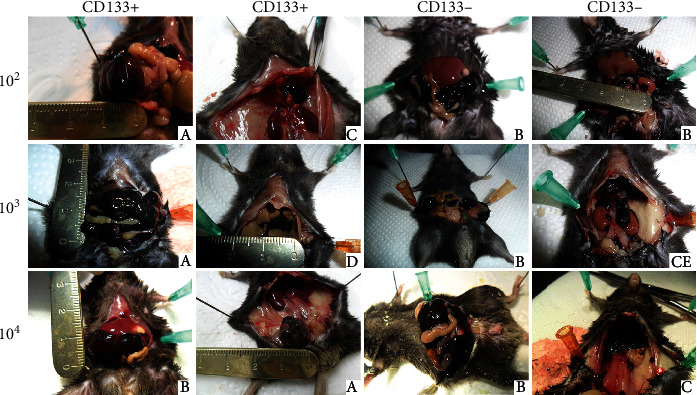
Tumor development depending on density and phenotype of implanted B16-F10 cells. Examples of tumor placement: (a) peritoneum at the injection site, (b) intestinal mesentery, (c) diaphragm, (d) liver, and (e) kidneys.

Examples of tumor locations in the individual groups were presented in Figure 4. All tumors showed characteristic black color associated with the melanin accumulation. Tumors formed at the site of implantation, and those located in the intestinal mesentery reached considerable sizes. Tumors located in the area of organs such as the liver, kidneys, or diaphragm were smaller in size and different in shape (more oval).

### 3.6. No Differences in Tumor Mass, Necrosis Area, and Abnormal Mitosis Were Observed between Tested Populations

**Figure 5:**
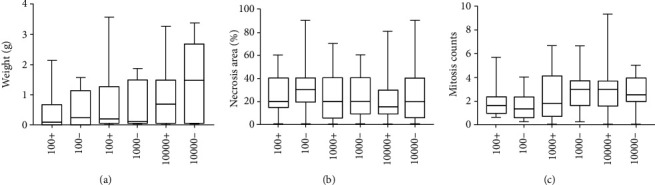
Tumor characteristic after end of follow-up: (a) average mass of tumors depending on the phenotype and number of implanted cells; (b) average values of necrosis areas in tumors depending on the phenotype and number of implanted cells; (c) average values of mitotic counts in tumors depending on the phenotype and number of implanted cells; (100+)—1 × 10^2^ B16-F10 cells expressing CD133 antigen implanted to mice; (100-)—1 × 10^2^ B16-F10 cells without expression of CD133 antigen implanted to mice.

The mean mass of tumors was the highest in the group of mice injected with 1 × 10^4^ B16-F10 CD133- cells and the lowest after the injection of 1 × 10^2^ CD133+ cells. There were no statistically significant differences in tumor mass between the tested groups of cells (*p* = 0.248). The graph of tumor mass dependence on the number and phenotype of implanted cells was shown in Figure 5(a). Average tumor mass was 0.74 g in CD133+ and 0.78 g in CD133- group (*p* = 0.877) (Figure S1).

Histopathological evaluation showed that all tested tumors were malignant melanomas. Areas of tumor necrosis were between 0 and 90%. The highest mean values of necrosis areas in the isolated tumors were observed in the group of mice injected with 1 × 10^2^ CD133- cells and the lowest in mice injected with 1 × 10^3^ and 1 × 10^4^ CD133- cells. Moreover, the necrosis area did not differ significantly between the compared groups (*p* = 0.676). The relationship between the fraction and the number of implanted cells and the mean value of necrosis areas was shown in Figure 5(b).

In all tumors, numerous and abnormal mitoses were found (Figure 5(c), Fig. S2). In the groups in which 1 × 10^3^ of cells were implanted, the highest number of tumors with a mitotic index above 5 was observed (Table 1). There were no statistically significant differences between all studied groups (*p* = 0.239).

## 4. Discussion

Malignant melanoma is one of the most aggressive type of cancers, which incidence in recent years is rapidly increasing [14]. One of the main problems in melanoma treatment is aggressiveness of tumor and ability to metastasis [15]. Limited effect of therapy in patients with advanced disease suggests that melanoma contains large number of cells resistant to systemic treatment. This concept justifies the presence of CSCs that can play an important role in cancer recurrence and metastasis [10, 16]. CSC conception assumes that tumor can be developed from a single CSC and that is why all of them have to be eradicated during therapy. Many studies are dealing with development of an effective method for CSC identification, which in the future can open new treatment perspectives [17]. The aim of this study was an attempt to evaluate the suitability of CD133 surface marker for identification of melanoma CSCs.

Development of flow cytometry is allowed for detection of surface markers which in turn is allowed for CSC searching and identification. One of the first potentially detected CSCs was CD34+ CD38- myeloid leukemia cells [18]. In solid tumors, first cancer stem cells were described as CD44 + CD24−/low lineage− in a breast cancer [19]. CD133 was at first identified as a marker of hematopoietic progenitor cells and nerve stem cells, but the presence of this marker was also detected in many other cancers including melanoma [20, 21].

CSCs represent very small population (<1%) of whole melanoma cells, which is consistent with the results obtained in this study (0.12%) [21, 22]. We used magnetic cell sorting for CD133+ melanoma cell isolation from B16-F10 cell line. Similar technique was successfully used for isolation of the same cell population from other melanoma cells like BLM, MV3, and A375 [23–25]. In our experiment, we observed the differences in cell proliferation between CD133+ cells and two other tested populations. This difference was especially visible when higher cell densities were used for analysis. In other study, it was shown that cells expressing both CD133 and CD44 antigens create a vascular niche in melanoma xenografts [26]. 2D cell culture *in vitro* forces a different growth characteristic compared to *in vivo* conditions due to exit of stem cells from the niche microenvironment. For cells growing on a flat surface, very important is cell-to-cell contact, and this feature differs between cell types which means that some cell lines have to be seeded in higher density; otherwise, inhibition of proliferation due to lack of signals from neighboring cells might occur. It is well known that cells expressing CD133 marker lose it during division and differentiation, that is why even when CD133+ cells will be seeded alone after another isolation, we cannot obtain a higher number of positive cells [27]. CD133+ cells seeded in higher density divide faster and consequently differentiate giving larger numbers of CD133 negative cells restoring the pool of positive cells (Figure 2(b)). Lower density of CD133+ cells after the first division cycles restore pool of CD133+ cells similar to this in heterogeneous population and CD133- population in which positive cells appear probably as a result of dedifferentiation. That is why in this case, the growth characteristic of all three tested populations was similar (Figure 2(a)). Faster growth of CD133+ cells seeded in higher density was also caused by generation of the holoclones that are characterized by extensive proliferation and self-renewal potential [28]. Holoclones express survival and self-renewal genes associated with stem cell capacity [28]. Clonogenic assay performed in our study showed that in all tested densities, a higher number of holoclones was visible in the CD133+ group. Additionally, in the CD133- population, seeded in the lowest density, lack of holoclones was observed (Figure 3(b)). Our *in vitro* experiments showed that CD133+ melanoma cells are characterized by different properties compared to cells not expressing this marker. An advantage of CD133+ melanoma cells compared to CD133- and unsorted population, based on the obtained results, may indicate stem cell properties of CD133+. Melanoma cells expressing this marker showed increased chemoresistance for drugs like caffeic acid phenethyl ester, taxol, or fotemustine compared to CD133- cells [23–25].

Presence of CD133 as a stem cell marker is more complex and can vary between different melanoma cell lines. In the study of Zimmer et al., 9 different human melanoma cell lines were analyzed; percentage of CD133+ cells varied between 1 and 90%, but only positive cells isolated from D10 cell line possessed significantly higher clonogenic potential [29]. Melanoma cancer stem cell identification is probably even more complex, and for its proper detection, the analysis of coexpression of several markers is required. Higher clonogenic potential was observed in CD133+, CD44+, and CD24+ cells isolated from B16-F10 cell line [30]. Cells with this phenotype differ also in morphology (more rounded in appearance) while cells not expressing those three markers were spindle shaped. In our study, we did not observed changes in morphology between three tested populations, separated using only CD133 antigen, which support thesis that utilization of more than one marker for CSC isolation is necessary.

Our results showed that properties of CD133+ population observed *in vitro* were not confirmed *in vivo*. In the study of Monzani et al., only CD133+ isolated from human melanoma (expression level similar like in our study above 1%) after implantation to NOD/SCID mice in number 1 × 10^5^ induced palpable tumors after 40 days. Induction of tumor by CD133- cells was not visible even after 4-month observation [21]. In the other study performed on 9 different melanoma cell lines likewisely, only cells expressing CD133 marker induced tumor formation after implantation to SCID mice line [30]. Results presented by the other group, similarly to results presented in our study, showed that 27% of melanoma cells had ability of tumor formation. 50 different surface markers of human melanoma were analyzed from which 15 were selected (including CD133) as a potential procarcinogenic marker. Cells with different marker expression were implanted to the NOD/SCID and NOD/SCID Il2rg-/-. In the second mice strain characterized by higher degree of the immune system impairment, all implanted cells generated tumor growth [31]. Most of the studies dealing with CSC detection and identification come from xenograft research in which human cells are implanted to immunodeficient animals. The interpretation of these results may be difficult due to impact of the barrier between species, together with environmental and immunological interactions. In our study, cells were implanted to syngeneic animals which could be the reason of tumor generation by both analyzed melanoma populations. Together with the previously described study, our results indicate that for melanoma CSC identification analysis of coexpression of other markers is necessary. B16-F10 melanoma cells with expression of CD133, CD44, and CD24 generated the fastest tumor formation compared to cells with other phenotype, despite that they were implanted in five times smaller density [30]. Other potential markers that could be used for the identification of melanoma CSCs are members of ABC family (ABCG2, ABCB5), CD166, and nestin [21, 32, 33]. Interesting candidate is also CD271 surface marker (p75 neutropine) [34, 35]. The expression of CD133 marker was also observed in melanoma disseminated tumor cells (HMB-45+ and CD45-). Lack of CD45 antigen enables for discrimination of these cells in the bone marrow [36].

The place of cell injection also plays important role in tumor development. The study of Zhong et al. showed that injection of at least 1 × 10^4^ of B16-F10 cells intravenously or subcutaneously was necessary for tumor development in C57BL/6 mice, while only 10 cells were enough to form tumor after intraperitoneally implantation in 80% cases [37]. In our study, performed on the same animal model, intraperitoneally implantation of 1 × 10^2^ CD133+ and CD133- cells resulted in tumor formation, respectively, in 54% and 38% cases. Tumor growth after CD133 negative B16-F10 cell implantation was probably resulted by ability of this cell population to get stem cell properties by accumulation of genetic changes or by environmental factors and signals coming from the niche of the recipient's organism [38, 39]. Meta-analysis of melanoma patients' sections showed that CD133 alone is not a proper biomarker in the identification of melanoma CSCs. Analysis included 299 melanoma patients and was performed using immunohistochemical analysis [8]. Correlation between high CD133 expression and melanoma progression would be more significant when coexpression of two or more markers is studied.

## 5. Conclusions

Taking together, the expression of CD133 marker promotes B16-F10 melanoma cell growth *in vitro*. After implantation to the animal model, both CD133+ and CD133- populations led to tumor development. Analysis of coexpression of CD133 with other marker is necessary for proper identification of melanoma CSCs.

## Figures and Tables

**Table 1:** Tumor mitotic index (%) evaluation depending on the phenotype and number of the examined cells.

	1 × 10^2^			1 × 10^3^			1 × 10^4^		
Scale	0-3	3-5	>5	0-3	3-5	>5	0-3	3-5	>5
CD133+	40.9	4.6	4.6	36.2	8.5	6.4	42.4	18.2	3.0
CD133-	40.9	9.1	0	34.0	10.6	4.3	21.2	15.2	0

## Data Availability

All data were presented within the manuscript.

## Abbreviations

CSCs: Cancer stem cells
DMEM/Ham's F12 medium: Dulbecco's Modified Eagle Medium
FBS: Fetal bovine serum
MACS: Magnetic-activated cell sorting.

## Supplementary Materials

Supplementary 1 Figure S1. Distribution of tumor properties. Mean tumor mass, necrosis area, and mitotic counts were presented on violin graphs in order to illustrate its distribution between tested groups (CD133+ vs. CD133-).

Supplementary 2 Figure S2. Evaluation of mitotic index. Mitosis examples (arrow) in tumors depending on the phenotype and number of implanted cells, light microscope, bar = 50 *μ*m.

Supplementary 3 Table S1. Detailed distribution of tumorigenesis in vivo depending on the expression of the CD133 marker and the number of implanted cells.
