# Supplementary figures and images for: CD133 Antigen as a Potential Marker of Melanoma Stem Cells: In Vitro and In Vivo Studies
Source: Stem Cells Int. 2020 Dec 23;2020:8810476. doi: 10.1155/2020/8810476 (PMC7774302; doi:10.1155/2020/8810476)

CD133+

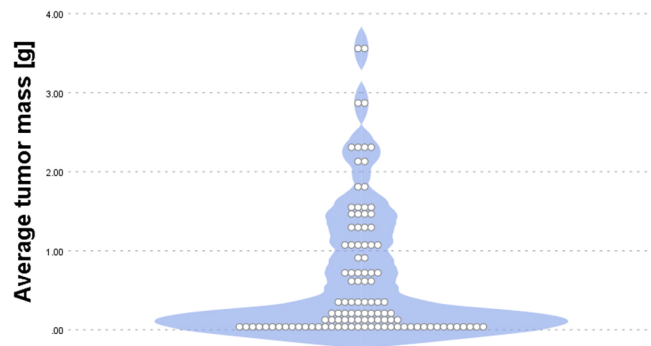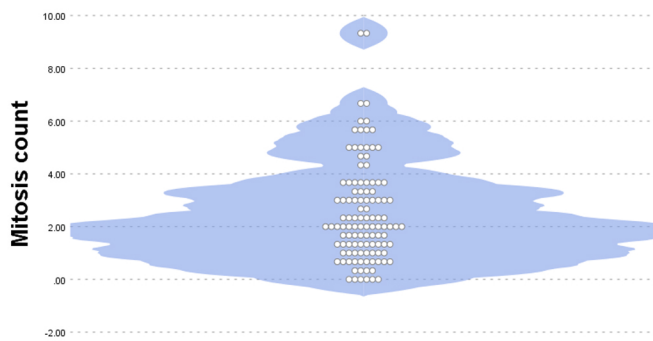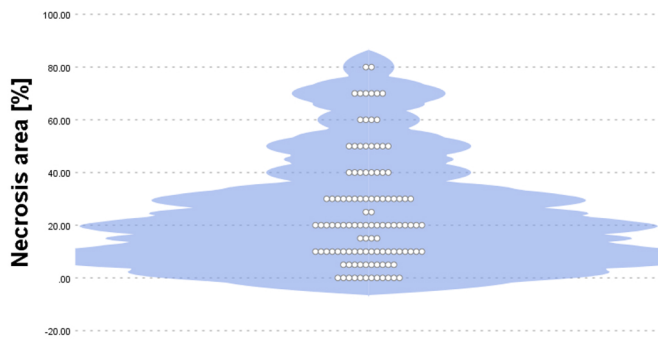

CD133-

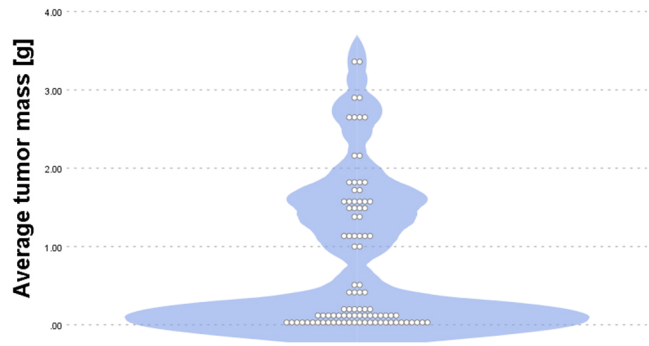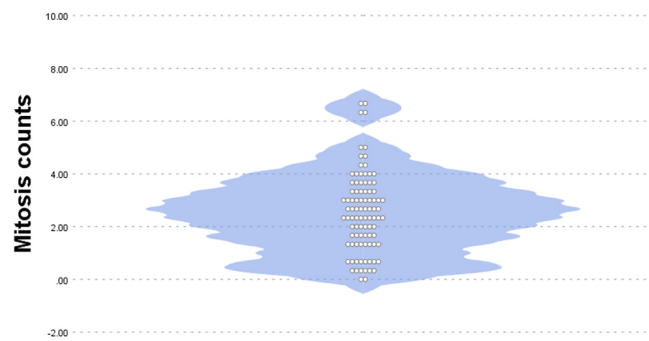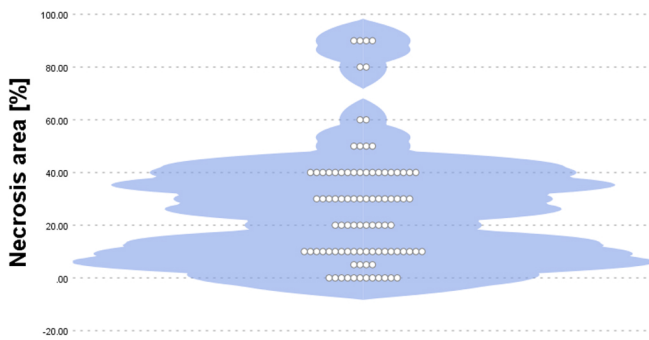

Supplement: Supplementary 1 — Figure S1. Distribution of tumor properties. Mean tumor mass, necrosis area, and mitotic counts were presented on violin graphs in order to illustrate its distribution between tested groups (CD133+ vs. CD133-). [file 8810476.f1.pdf]

CD133+

CD133-

100

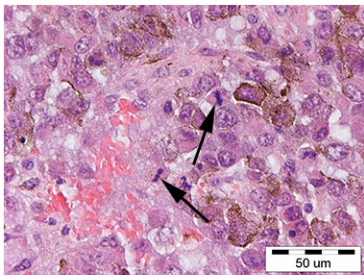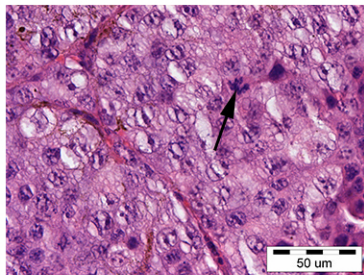

1000

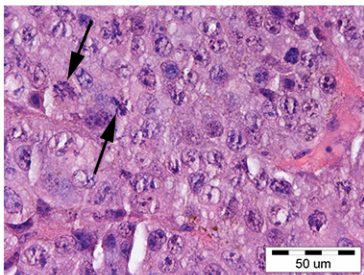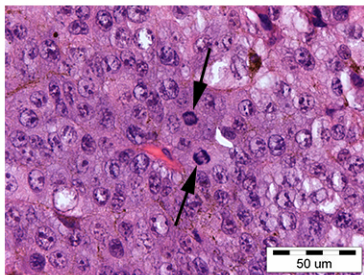

10 000

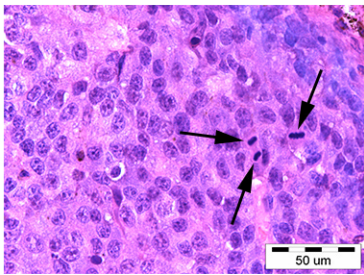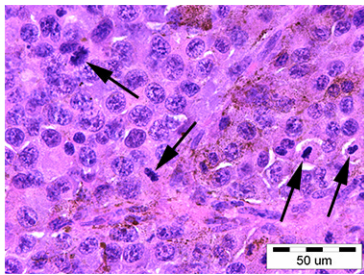

Supplement: Supplementary 2 — Figure S2. Evaluation of mitotic index. Mitosis examples (arrow) in tumors depending on the phenotype and number of implanted cells, light microscope, bar = 50 μm. [file 8810476.f2.pdf]
