# Supplementary material for: CD133 Antigen as a Potential Marker of Melanoma Stem Cells: In Vitro and In Vivo Studies
Source: Stem Cells Int. 2020 Dec 23;2020:8810476. doi: 10.1155/2020/8810476 (PMC7774302; doi:10.1155/2020/8810476)
Supplement: Supplementary 3 — Table S1. Detailed distribution of tumorigenesis in vivo depending on the expression of the CD133 marker and the number of implanted cells. [file 8810476.f3.docx]

Table S1. Detailed distribution of tumorigenesis in vivo depending on the expression of the CD133 marker and the number of implanted cells.

| Cells | Seminal vesicle | | | Peritoneum | | | Kidney | | | Liver | | | Diaphragm | | | Lung | | |  |
| --- | --- | --- | --- | --- | --- | --- | --- | --- | --- | --- | --- | --- | --- | --- | --- | --- | --- | --- | --- |
|  | Average  Weight[g]/  number of tumors | necrosis area [%] | mitosis counts | Average  Weight[g]/  number of tumors | necrosis area [%] | mitosis counts | Average  weight[g]/  number of tumors | necrosis area [%] | mitosis counts | Average  weight[g]/number of tumors | necrosis area [%] | mitosis counts | Average  weight[g]/  number of tumors | necrosis area [%] | mitosis counts | Average  weight[g]/  number of tumors | necrosis area [%] | mitosis counts | End of experiment  (days) |
| 10^2^+ | 0.04 (n=1) | 30 | 2.00 |  |  |  |  |  |  |  |  |  |  |  |  |  |  |  | 42 |
| 10^2^+ | 0.01 (n=1) | 0 | 1.00 | 0.65 (n=2) | 50 | 1.33 |  |  |  |  |  |  |  |  |  |  |  |  | 42 |
| 10^2^+ |  |  |  |  |  |  |  |  |  |  |  |  |  |  |  |  |  |  | 42 |
| 10^2^+ |  |  |  |  |  |  |  |  |  |  |  |  |  |  |  |  |  |  | 42 |
| 10^2^+ |  |  |  |  |  |  |  |  |  |  |  |  |  |  |  |  |  |  | 42 |
| 10^2^+ |  |  |  |  |  |  |  |  |  |  |  |  |  |  |  |  |  |  | 42 |
| 10^2^+ |  |  |  |  |  |  |  |  |  |  |  |  |  |  |  |  |  |  | 42 |
| 10^2^+ |  |  |  |  |  |  |  |  |  |  |  |  | 0.13 (n=1) | 5 | 3.67 |  |  |  | 42 |
| 10^2^+ |  |  |  | 1.04 (n=4) | 60 | 1.00 | 0.09 (n=1) | 40 | 2.00 |  |  |  |  |  |  |  |  |  | 42 |
| 10^2^+ |  |  |  |  |  |  |  |  |  |  |  |  |  |  |  |  |  |  | 42 |
| 10^2^+ |  |  |  | 2.13 (n=4) | 25 | 0.67 |  |  |  |  |  |  | 0.06 (n=1) | 15 | 1.67 |  |  |  | 20 |
| 10^2^+ |  |  |  | 0.34(n=12) | 20 | 1.67 |  |  |  |  |  |  | 0.08 (n=8) | 20 | 5.67 |  |  |  | 21 |
| 10^2^+ |  |  |  | 0.05 (n=1) | 20 | 2.33 |  |  |  |  |  |  |  |  |  |  |  |  | 42 |
|  |  |  |  |  |  |  |  |  |  |  |  |  |  |  |  |  |  |  | |
| 10^3^+ |  |  |  |  |  |  |  |  |  |  |  |  |  |  |  |  |  |  | 42 |
| 10^3^+ |  |  |  | 0.01 (n=1) | 0 | to small |  |  |  |  |  |  |  |  |  |  |  |  | 42 |
| 10^3^+ |  |  |  |  |  |  |  |  |  |  |  |  |  |  |  |  |  |  | 42 |
| 10^3^+ |  |  |  |  |  |  |  |  |  |  |  |  |  |  |  |  |  |  | 42 |
| 10^3^+ |  |  |  |  |  |  |  |  |  |  |  |  |  |  |  |  |  |  | 42 |
| 10^3^+ |  |  |  | 1.29 (n=2) | 30 | 3.00 | 0.19 (n=1) | 5 | 1.00 | 0.16 (n=1) | 20 | 2.67 |  |  |  |  |  |  | 14 |
| 10^3^+ |  |  |  | 1.57 (n=2) | 10 | 1.33 | 0.11 (n=1) | 20 | 0.33 |  |  |  | 0.04 (n=1) | 70 | 0 |  |  |  | 16 |
| 10^3^+ |  |  |  | 2.33 (n=1) | 50 | 3.33 | 0.07 (n=1) | 40 | 0.67 | 0.06 (n=1) | 5 | 1.00 |  |  |  |  |  |  | 17 |
| 10^3^+ |  |  |  | 2.29 (n=1) | 5 | 1.67 | 0.05 (n=1) | 30 | 0.33 |  |  |  | 0.03 (n=3) | 20 | 2.00 |  |  |  | 17 |
| 10^3^+ |  |  |  | 3.56 (n=1) | 40 | 6.00 | 0.22 (n=3) | 30 | 6.67 | 0.39 (n=1) | 30 | 2.00 | 0.05 (n=5) | 10 | 1.33 |  |  |  | 15 |
| 10^3^+ |  |  |  | 1.11 (n=3) | 20 | 5.00 | 0.33 (n=2) | 70 | 2.00 |  |  |  |  |  |  |  |  |  | 16 |
| 10^3^+ |  |  |  | 0.72 (n=5) | 50 | 4.33 | 0.32 (n=2) | 10 | 5.67 | 0.16 (n=3) | 0 | 1.67 |  |  |  |  |  |  | 16 |
| 10^3^+ |  |  |  | 1.82 (n=2) | 40 | 4.67 | 0.18 (n=3) | 0 | 0.67 |  |  |  |  |  |  |  |  |  | 16 |

Table S1. Detailed distribution of tumorigenesis in vivo depending on the expression of the CD133 marker and the number of implanted cells.

| 10^4^+ | 0.06 (n=1) | 20 | 0.67 |  |  |  |  |  |  | 0.04 (n=4) | 50 | 0.67 | 0.02 (n=1) | 30 | 3.00 |  |  |  | 17 |
| --- | --- | --- | --- | --- | --- | --- | --- | --- | --- | --- | --- | --- | --- | --- | --- | --- | --- | --- | --- |
| 10^4^+ |  |  |  |  |  |  |  |  |  |  |  |  |  |  |  |  |  |  | 42 |
| 10^4^+ |  |  |  | 1.54 (n=2) | 70 | 2.00 |  |  |  |  |  |  |  |  |  |  |  |  | 42 |
| 10^4^+ |  |  |  | 0.06 (n=1) | 10 | 2.33 |  |  |  |  |  |  |  |  |  |  |  |  | 42 |
| 10^4^+ | 0.01 (n=1) | 20 | 3.67 | 1.30 (n=2) | 15 | 2.33 |  |  |  |  |  |  | 0.03 (n=2) | 80 | 1.33 |  |  |  | 17 |
| 10^4^+ | 0.15 (n=1) | 0 | 1.33 | 0.92 (n=3) | 10 | 3.67 |  |  |  | 0.01 (n=1) | 10 | 5.00 |  |  |  |  |  |  | 14 |
| 10^4^+ |  |  |  | 1.49 (n=2) | 60 | 3.00 |  |  |  |  |  |  |  |  |  |  |  |  | 14 |
| 10^4^+ |  |  |  | 2.87 (n=1) | 5 | 3.00 |  |  |  |  |  |  |  |  |  |  |  |  | 14 |
| 10^4^+ |  |  |  | 1.44 (n=2) | 20 | 0.00 |  |  |  |  |  |  |  |  |  |  |  |  | 15 |
| 10^4^+ |  |  |  | 0.69 (n=5) | 30 | 3.67 |  |  |  | 0.58 (n=1) | 10 | 2.00 |  |  |  |  |  |  | 15 |
| 10^4^+ |  |  |  | 0.75 (n=4) | 30 | 9.33 |  |  |  |  |  |  | 0.23(n=11) | 0 | 2.33 |  |  |  | 16 |
| 10^4^+ |  |  |  | 1.10 (n=3) | 10 | 3.33 |  |  |  | 0.20 (n=1) | 10 | 3.00 |  |  |  |  |  |  | 16 |
| 10^4^+ |  |  |  | 3.25 (n=1) | 10 | 5.00 |  |  |  |  |  |  |  |  |  |  |  |  | 16 |
|  |  |  |  |  |  |  |  |  |  |  |  |  |  |  |  |  |  |  | |
| 10^2^- |  |  |  |  |  |  |  |  |  |  |  |  |  |  |  |  |  |  | 42 |
| 10^2^- |  |  |  |  |  |  |  |  |  |  |  |  |  |  |  |  |  |  | 42 |
| 10^2^- |  |  |  |  |  |  |  |  |  |  |  |  |  |  |  |  |  |  | 42 |
| 10^2^- |  |  |  |  |  |  |  |  |  |  |  |  |  |  |  |  |  |  | 42 |
| 10^2^- |  |  |  |  |  |  |  |  |  |  |  |  |  |  |  |  |  |  | 42 |
| 10^2^- |  |  |  |  |  |  |  |  |  |  |  |  |  |  |  |  |  |  | 42 |
| 10^2^- |  |  |  |  |  |  |  |  |  |  |  |  |  |  |  |  |  |  | 42 |
| 10^2^- |  |  |  |  |  |  |  |  |  |  |  |  |  |  |  |  |  |  | 42 |
| 10^2^- |  |  |  | 1.11 (n=6) | 90 | 1.33 |  |  |  |  |  |  |  |  |  |  |  |  | 42 |
| 10^2^- |  |  |  | 1.16 (n=6) | 40 | 0.33 |  |  |  | 0.23 (n=2) | 30 | 0.33 |  |  |  |  |  |  | 22 |
| 10^2^- | 0,42 (n=4) | 40 | 4.00 | 0.02 (n=1) | 10 | 2.33 | 0.01 (n=2) | 40 | 2.33 |  |  |  | 0.03 (n=1) | 20 | 2.00 | 0.01 (n=1) | 0 | 1.33 | 42 |
| 10^2^- |  |  |  | 0.51 (n=7) | 30 | 1.33 |  |  |  |  |  |  |  |  |  |  |  |  | 42 |
| 10^2^- |  |  |  | 1.55 (n=2) | 40 | 0.67 | 0.03 (n=3) | 20 | 3.33 |  |  |  |  |  |  |  |  |  | 22 |

Table S1. Detailed distribution of tumorigenesis in vivo depending on the expression of the CD133 marker and the number of implanted cells.

| 10^3^- |  |  |  |  |  |  |  |  |  |  |  |  |  |  |  |  |  |  | 42 |
| --- | --- | --- | --- | --- | --- | --- | --- | --- | --- | --- | --- | --- | --- | --- | --- | --- | --- | --- | --- |
| 10^3^- |  |  |  |  |  |  |  |  |  |  |  |  |  |  |  |  |  |  | 42 |
| 10^3^- |  |  |  |  |  |  |  |  |  |  |  |  |  |  |  |  |  |  | 42 |
| 10^3^- |  |  |  |  |  |  |  |  |  |  |  |  |  |  |  |  |  |  | 42 |
| 10^3^- |  |  |  |  |  |  |  |  |  |  |  |  |  |  |  |  |  |  | 42 |
| 10^3^- | 0.41 (n=2) | 20 | 6.67 | 1.85 (n=2) | 40 | 6.33 |  |  |  | 0.17 (n=3) | 10 | 4.00 | 0.09 (n=3) | 40 | 1.67 | 0.01 (n=1) | 0 | 0.33 | 14 |
| 10^3^- |  |  |  | 1.47 (n=3) | 50 | 2.67 | 0.14 (n=2) | 20 | 2.67 |  |  |  | 0.01 (n=1) | 0 | 0.67 |  |  |  | 14 |
| 10^3^- |  |  |  | 1.51 (n=3) | 10 | 4.33 |  |  |  |  |  |  | 0.04 (n=1) | 10 | 0.67 |  |  |  | 15 |
| 10^3^- |  |  |  | 1.60 (n=2) | 20 | 2.67 | 0.12 (n=1) | 60 | 3.00 | 0.12 (n=5) | 30 | 1.67 | 0.06 (n=3) | 0 | 3.00 |  |  |  | 17 |
| 10^3^- |  |  |  | 1.79 (n=2) | 30 | 3.33 | 0.09 (n=2) | 40 | 2.33 |  |  |  | 0.02 (n=1) | 30 | 0.67 |  |  |  | 15 |
| 10^3^- |  |  |  | 1.11 (n=3) | 50 | 3.67 | 0.10 (n=2) | 10 | 3.00 |  |  |  | 0.15 (n=1) | 10 | 1.67 |  |  |  | 16 |
| 10^3^- |  |  |  | 1.72 (n=2) | 30 | 3.67 |  |  |  | 0.11 (n=5) | 10 | 3.00 | 0.05 (n=1) | 0 | 3.00 |  |  |  | 16 |
| 10^3^- |  |  |  |  |  |  |  |  |  |  |  |  |  |  |  |  |  |  | 42 |
|  |  |  |  |  |  |  |  |  |  |  |  |  |  |  |  |  |  |  | |
| 10^4^- |  |  |  |  |  |  |  |  |  |  |  |  |  |  |  |  |  |  | 42 |
| 10^4^- |  |  |  | 0.21 (n=1) | 80 | 2.00 |  |  |  |  |  |  |  |  |  |  |  |  | 42 |
| 10^4^- |  |  |  |  |  |  |  |  |  |  |  |  |  |  |  |  |  |  | 42 |
| 10^4^- |  |  |  | 0.01 (n=1) | 90 | 0 |  |  |  |  |  |  |  |  |  |  |  |  | 42 |
| 10^4^- |  |  |  |  |  |  |  |  |  |  |  |  |  |  |  |  |  |  | 42 |
| 10^4^- |  |  |  | 2.16 (n=1) | 0 | 5.00 |  |  |  |  |  |  |  |  |  |  |  |  | 14 |
| 10^4^- |  |  |  | 2.90 (n=1) | 40 | 4.67 |  |  |  |  |  |  |  |  |  |  |  |  | 14 |
| 10^4^- |  |  |  | 1.38 (n=2) | 10 | 2.33 |  |  |  |  |  |  |  |  |  |  |  |  | 15 |
| 10^4^- |  |  |  | 2.67 (n=1) | 5 | 2.67 |  |  |  | 0.01 (n=1) | 40 | 1.33 |  |  |  |  |  |  | 16 |
| 10^4^- |  |  |  | 2.63 (n=1) | 10 | 3.67 |  |  |  |  |  |  |  |  |  |  |  |  | 14 |
| 10^4^- |  |  |  | 1.00 (n=3) | 30 | 3.33 |  |  |  |  |  |  |  |  |  |  |  |  | 15 |
| 10^4^- |  |  |  | 3.36 (n=1) | 30 | 4.00 |  |  |  | 0.03 (n=4) | 10 | 2.33 |  |  |  |  |  |  | 17 |
| 10^4^- |  |  |  | 1.56 (n=2) | 5 | 2.00 |  |  |  |  |  |  |  |  |  |  |  |  | 17 |
